# Beyond the common metrics: Expanding the impact of the KL2 mentored career development program using alternative impact assessment frameworks

**DOI:** 10.1017/cts.2019.375

**Published:** 2019-07-11

**Authors:** Beatrice A. Boateng, Nia Indelicato, Ellen P. Fischer, Pedro L. Delgado, Mary E. Aitken, Laura P. James

**Affiliations:** 1 Translational Research Institute, University of Arkansas for Medical Sciences, Little Rock, AR, USA; 2 Department of Pediatrics, University of Arkansas for Medical Sciences, Little Rock, AR, USA; 3 Department of Psychiatry, University of Arkansas for Medical Sciences, Little Rock, AR, USA; 4 Arkansas Children’s Research Institute, Little Rock, AR, USA

**Keywords:** CTSA, common metrics, impact assessment, KL2 program

The CTSA consortium has developed a career development Common Metric to address the impact of the KL2 training program. We argue that the current Common Metrics are limited and utilizing an expanded framework could provide a more holistic view of the impact of the KL2 training program.

Recognizing significant shortages in the translational research workforce [1], the National Institutes of Health (NIH) has funded various workforce development training programs and training grant mechanisms. The assessment of the outcomes of these efforts, including the program and trainee impact, has been a focus of considerable recent attention [2–10]. The KL2 Scholars Mentored Career Development Program provides protected research time to junior investigators and prepares them to transition to independent research funding.

Data to fully assess the impact of the KL2 career development program has been limited. In 2013, the Institute on Medicine report called for standard cross-CTSA metrics to assess the extent to which the CTSAs facilitate translational research [11]. The Common Metrics Workgroup was formed to identify, operationalize, and assess the feasibility of collecting Common Metrics [12]. The workgroup developed Careers in Clinical & Translational Research metrics focusing on the KL2 and TL1 programs to measure the number of CTSA hub-supported scientists that were continuously engaged in clinical and translational research after the training. This measure was designed to assess long-term value of the investment made in the scholars and the sustainability of research careers for trainees. While the Common Metrics for career development seek information on the impact of the consortium in the training and retention of translational researchers, additional appraisal of the implementation of research outcomes is needed to fully understand the return on investment in research. The focus on publications and grants as the primary outcomes of research training is too narrow and increasingly less relevant in the modern era [5].

A recent systematic review of methodological frameworks for the assessment of the impact of biomedical research identified five broad categories of impact: (1) primary research-related, (2) policymaking, (3) health and health systems, (4) health-related and societal, and (5) broader economic impact [13]. This broader set of outcome categories has not uniformly been applied to assess the impact of the research of early career biomedical scientists and those trained in career development programs.

## The Process: How We Did It?

In 2015, we created a semi-structured interview guide, using the Becker list for Assessment of Research Impact [14] as a framework for assessing research and scholarship impact. Similar to other research impact frameworks, the Becker list attempts to capture important but lesser known aspects of research and track post-publication impact of an individual’s research in five categories: advancement of knowledge, clinical implementation, community benefit, legislation and policy, and economic benefit. The model provides a systematic approach to identifying researcher collaborations, how research findings are being used, and how research has benefitted intended populations. The interview guide included 60 impact indicators on the Becker list. Initial pilot testing of the instrument was performed on two graduates of the KL2 program, to assess the feasibility of utilizing all 60 indicators and the time needed to administer it. The average length of time to complete the interview was approximately an hour. This process revealed redundancy in some of the items asked and, through an iterative process, we identified and eliminated (1) items in the initial instrument that could be collected electronically such as publications and funding, (2) other productivity data collected routinely in quarterly scholar reports, and (3) duplicative items. Through this exercise, we reduced the interview guide to 13 items. The final instrument (Appendix A) has two parts. Part A includes a summary of the KL2 Scholar’s research, research products (devices, drugs, procedures, questionnaires/instruments, grants, publications, etc.), and research collaborations, as documented in their quarterly reports. Data was also gleaned from electronic sources (publication citations) to make it easier on the scholar and assure greater precision in the data collected. Part B is the semi-structured 13-item interview guide. To examine the utility of the modified instrument, we tested it with graduates of our KL2 Scholars Mentored Career Development Program, who had completed the program 3 years earlier and remained actively engaged in clinical and translational research. Three years was chosen to allow assessment of both short- and medium-term research impacts. This study received approval from our institutional research review board. After verifying the information obtained through the annual reports (data obtained for Part A), the scholar graduate was interviewed on their areas of impact based on the categories outlined in the Becker list (Part B). An “other” category was included to address activities that did not fit into any of the five categories. We adopted the thematic analysis approach for analyzing the data. First, the interview questions that received an affirmative response were identified and tallied for each scholar to identify their area of impact. Affirmative responses were also verified by investigating evidence through the annual reports, the interview, or requesting evidence from the scholar.

### Participants

Of the 24 scholars who had participated in the KL2 program since 2009, 11 were eligible for participation in the study, excluding the 2 graduates who participated in the initial testing of the instrument. These scholars were demographically diverse: six females and five males; and three African Americans, four Asian, and four Caucasian. Participation in this impact evaluation was voluntary. Of the 11 eligible for the study, we interviewed 6 scholars within the data collection time frame.

## Areas of Impact

**Fig. 1.**
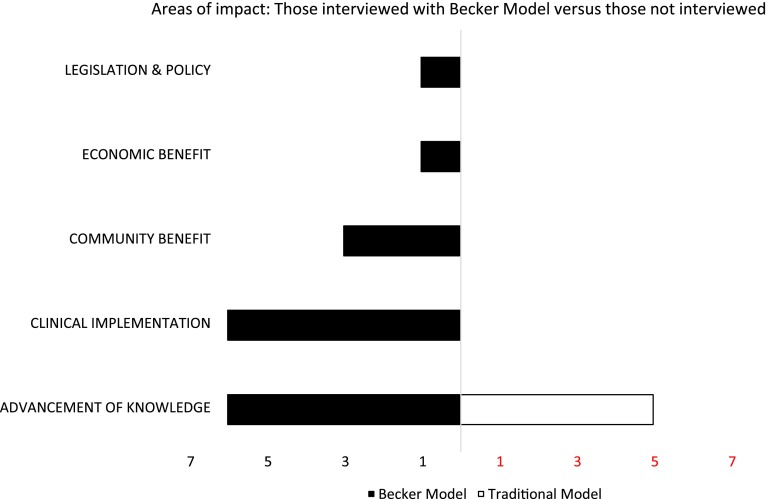
Areas of impact of those interviewed with Becker list (6) versus those not interviewed (5).

Based on the categories of the Becker list, all eligible participants (N = 11) had impact in the advancement of knowledge category, which includes outputs that contribute to the scholarly record of the scholar such as publications. Five of the 11 were not interviewed, so we were unable to determine any further areas of impact beyond this category. Of the six who were interviewed, we identified additional impact in the areas of clinical implementation, community benefit, legislation and policy, and economic benefit (Fig. 1). All the research conducted by the six interviewed scholars had a clinical implementation component, which included recruitment of patients and adoption of clinical approaches to improve outcomes. Three of the participants conducted research that benefited communities, particularly in the area of mental health in underrepresented communities. This information was gleaned from the open-ended responses.

Of note, these three participants were women from underrepresented groups. One scholar, who had impact in the area of legislation and policy and economic benefit, had a mentor with extensive experience in influencing legislation within the state. While we were able to verify some of the information through the reports and publications, we were unable to quantify the policy or the economic benefit impact.

## Implications

There is increasing interest to assessing the impact of training programs such as the KL2 Scholars Mentored Career Development Program to demonstrate the value and return on research investments. To date, academic-oriented outputs such as peer-reviewed articles, the h-index, citation rates, and the number of grants are the most widely utilized indicators to measure research impact. Assessing impact under the academic realm drives us to lean toward more easily identifiable activities and limits our purview of other areas of impact beyond academia. There is a selection bias toward more obvious outputs. Using the Becker list, this impact analysis demonstrated substantial value of scholars’ research that is overlooked using only conventional metrics. For instance, the careers in clinical and translational Common Metric includes the impact on women and underrepresented minorities, but only offers a count of individuals who have participated in the training and does not address impact of their research in general or on underrepresented populations. Though limited to one institution, our findings demonstrate that there is value in using a more expanded impact assessment framework for career development. The data reported is based on the work of a subset of scholars who participated in the program between 2010 and 2014; results therefore likely underestimate the true outputs of those interviewed. A larger study, inclusive of all the KL2 programs in the CTSA consortium, could potentially fill in the gaps not addressed by the Common Metrics. Similar to other studies [5,15], we argue that additional measures of impact be considered.

This alternative approach is, however, not without limitations. The current Common Metrics are readily accessible. Expanding the measures of impact using our interview guide requires a brief interview and continuous assessment to address the time it takes to be impactful in a field. Using a variety of indicators and not limiting impact to academic indicators could provide a more holistic view of the impact of training programs.

## Appendix A Instrument Used

### PART A: Data Collected Through the Scholar’s Record and Quarterly Reports

#### Name of KL2 Scholar:

1. Years in program:
2. Areas of research:
3. Did you use the Institutional Animal Care and Use Committee: (Yes/No)
4. Did your research require an Investigational New Drug exemption application (Yes/No)
5. Did your research require an investigational device exemption application (Yes/No)

#### Scholarly Outputs

- Summary of Scholar’s research (from quarterly reports)
- **Research products:** Publications, Grants, Patents, Questionnaires, instruments, Honors/Awards, Devices, Drugs, Procedures, Other

### PART B: Semi-Structured Interview Guide

**Table:**

	Key				
	AK	CI	C	LP	EB
1. Did you recruit participants for your study? (yes/no)		X			
If yes, expand (race, age, disease)					
2. Did you partner with any community groups for your study? (yes/no)			X		
If yes, expand (disease specific, race)					
3. Did you develop a new investigational drug? (yes/no)	X				
If yes, expand If yes, has the application been accepted by the FDA? Has it shown any benefits?					
4. Did you develop any mobile applications/software as a result of your work (yes/no)	X				
If yes, name of app or software If yes, expand (who used your app, age group)					
5. Did you conduct any clinical trials (as a scholar or after completing the KL2)? (yes/no)		X			
If yes, expand on the trial					
6. Did you develop any algorithms or instrument as a result of the research you did as a KL2 scholar? (yes/no)		X			
If yes, expand Is anyone using algorithm or instrument?					
7. Did your research study findings result in the development of materials to assist with healthcare decision-making? (yes/no)			X		
If yes, expand					
8. Was a biological material application generated by the research study? (yes/no)	X				
If yes, expand					
9. Has your research been used in the development of guidelines? (yes/no)				X	
If yes, expand					
10. Has your research been used or cited in a policy development? (yes/no)				X	
If yes, expand					
Were you invited to testify based on your research? (yes/no)				X	
If yes, expand Were any changes made as a result of your testimony?					
Did your research result in cost reduction of health care delivery costs? (yes/no)					X
If yes, expand					
Did your research result in a cost-effective intervention for a condition? (yes/no)					X
If yes, expand					

Did your research result in anything else not mentioned here?

KEY: AK, advancement of knowledge; CI, clinical implementation; C, community benefit; LP, legislation and policy; EB, economic benefit.
